# *Bordetella* Adenylate Cyclase Toxin Inhibits Monocyte-to-Macrophage Transition and Dedifferentiates Human Alveolar Macrophages into Monocyte-like Cells

**DOI:** 10.1128/mBio.01743-19

**Published:** 2019-09-24

**Authors:** Jawid Nazir Ahmad, Jana Holubova, Oldrich Benada, Olga Kofronova, Ludek Stehlik, Martina Vasakova, Peter Sebo

**Affiliations:** a Laboratory of Molecular Biology of Bacterial Pathogens, Institute of Microbiology of the CAS, Prague, Czech Republic; b Laboratory of Molecular Structure Characterization, Institute of Microbiology of the CAS, Prague, Czech Republic; c Department of Respiratory Medicine, Thomayer Hospital, Prague, Czech Republic; Pasteur Institute

**Keywords:** *Bordetella*, adenylate cyclase toxin, cyclic AMP, dedifferentiation, macrophage

## Abstract

Monocytes arriving at the site of infection differentiate into functional effector macrophages to replenish the resident sentinel cells. Bordetella pertussis, the pertussis agent, secretes an adenylate cyclase toxin-hemolysin (CyaA) that binds myeloid phagocytes through complement receptor 3 (CD11b/CD18) and swiftly delivers its adenylyl cyclase enzyme domain into phagocytes. This ablates the bactericidal capacities of phagocytes through massive and unregulated conversion of cytosolic ATP into the key signaling molecule cAMP. We show that exposure of primary human monocytes to as low a concentration as 22.5 pM CyaA, or a low (2:1) multiplicity of infection by CyaA-producing B. pertussis bacteria, blocks macrophage colony-stimulating factor (M-CSF)-driven differentiation of monocytes. CyaA-induced cAMP signaling mediated through the activity of protein kinase A (PKA) efficiently blocked expression of macrophage markers, and the monocytes exposed to 22.5 pM CyaA failed to acquire the characteristic intracellular complexity of mature macrophage cells. Neither M-CSF-induced endoplasmic reticulum (ER) expansion nor accumulation of Golgi bodies, mitochondria, or lysosomes was observed in toxin-exposed monocytes, which remained small and poorly phagocytic and lacked pseudopodia. Exposure to 22.5 pM CyaA toxin provoked loss of macrophage marker expression on *in vitro* differentiated macrophages, as well as on primary human alveolar macrophages, which appeared to dedifferentiate into monocyte-like cells with upregulated CD14 levels. This is the first report that terminally differentiated tissue-resident macrophage cells can be dedifferentiated *in vitro*. The results suggest that blocking of monocyte-to-macrophage transition and/or dedifferentiation of the sentinel cells of innate immunity through cAMP-elevating toxin action may represent a novel immune evasion strategy of bacterial pathogens.

## Abstract (precis)

Macrophages are key sentinel cells of the immune system, and, as such, they are targeted by the toxins produced by the pertussis agent Bordetella pertussis. The adenylate cyclase toxin (CyaA) mediates immune evasion of B. pertussis by suspending the bactericidal activities of myeloid phagocytes. We reveal a novel mechanism of potential subversion of host immunity, where CyaA at very low (22 pM) concentrations could inhibit maturation of human monocyte precursors into the more phagocytic macrophage cells. Furthermore, exposure to low CyaA amounts has been shown to trigger dedifferentiation of mature primary human alveolar macrophages back into monocyte-like cells. This unprecedented capacity is likely to promote survival of the pathogen in the airways, both by preventing maturation of monocytes attracted to the site of infection into phagocytic macrophages and by dedifferentiation of the already airway-resident sentinel cells.

## INTRODUCTION

The repeat in toxin (RTX) family adenylate cyclase (AC) toxin (CyaA or ACT or AC-Hly) plays a key role in virulence of the whooping cough agent Bordetella pertussis (1, 2). CyaA is a 1,706-residue-long bifunctional toxin that delivers a cell-invasive N-terminal AC enzyme domain (384 residues) into host cells by the action of an RTX hemolysin/cytolysin moiety (1). CyaA binds the complement receptor 3 (known as CR3, α_M_β_2_ integrin, CD11b/CD18, and MAC-1) of host myeloid phagocytes such as monocytes, macrophages, neutrophils, and dendritic cells (3). The toxin inserts directly into the plasma membrane of cells to open a transient path for entry of extracellular calcium ions, which provokes a lateral relocation of the CyaA-CD11b/CD18 complex into cholesterol-enriched membrane lipid rafts and delivery of the AC enzyme domain directly into the cytosol of cells (4, 5). Upon activation by intracellular calmodulin, the AC enzyme catalyzes massive and unregulated conversion of ATP to the key signaling molecule cAMP (6, 7), which annihilates the bactericidal innate immune functions of phagocytes (8–11). Indeed, the synergy of CyaA/cAMP signaling through protein kinase A (PKA) and the exchange protein directly activated by cAMP (Epac) instantaneously blocks the superoxide burst of neutrophils, inhibits induction of nitric oxide production by macrophages, and hampers the complement- and Fc receptor-mediated opsonophagocytic uptake and killing of bacteria by the phagocytes (9–11). Finally, above a threshold concentration (100 pM), the CyaA toxin activity can trigger macrophage apoptosis (3, 12, 13).

Infection triggers production of chemoattractive cytokines and chemokines that induce monocyte extravasation and recruitment to the site of infection, where the produced cytokines orchestrate the differentiation of inflammatory monocytes into phagocytic and antigen-presenting macrophage cells. This is accompanied by acquisition of characteristic macrophage features, such as upregulated fatty acid synthesis, enlargement of macrophage cells accompanied by an increase in the numbers of organelles involved in bactericidal functions, expression of a number of surface receptors (e.g., CD36, CD206, CD11b, major histocompatibility complex class II [MHC II], CD204, CHIT1, and CHI3L1) and enhancement of the phagocytic capacity of macrophage cells (14), thus arming them for efficient clearing of pathogens.

Recently, we observed that action of the CyaA toxin accounted for massive infiltration of inflammatory myeloid cells into B. pertussis-infected mouse lung tissue and that these cells expressed reduced levels of MHC II molecules (15). This indicated that CyaA-produced cAMP signaling might hamper the phenotypic maturation of inflammatory monocytes in B. pertussis*-*infected tissue. We thus examined the impact of prolonged exposure of primary human blood monocytes to low (22.5 pM) CyaA toxin concentrations that do not provoke cell death but still increase intracellular cAMP levels and hijack cellular signaling. We show that the action of subcytotoxic amounts of CyaA toxin and a low multiplicity of infection (MOI) by CyaA-secreting B. pertussis bacteria block macrophage colony-stimulating factor (M-CSF)-induced maturation of primary human monocytes into macrophage cells *in vitro.* Moreover, we show for the first time that action of a bacterial toxin can trigger apparent dedifferentiation of terminally differentiated human alveolar macrophages.

## RESULTS

### CyaA toxin-produced cAMP signaling through protein kinase A inhibits differentiation of monocytes into macrophages.

**FIG 1:**
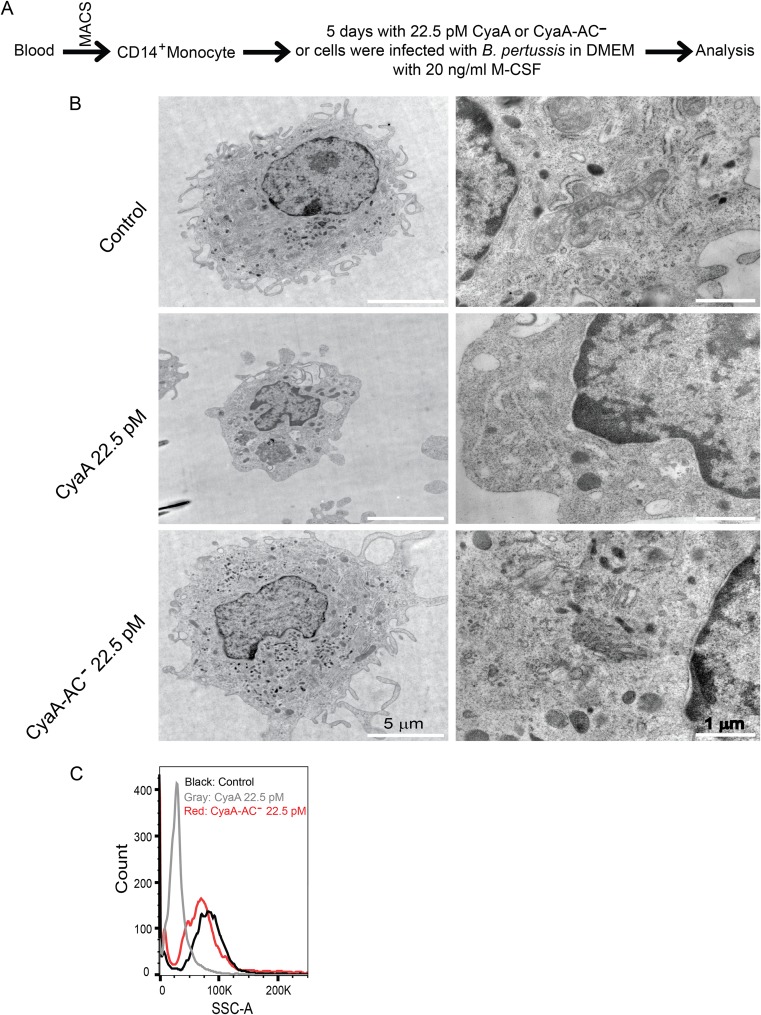
CyaA subverts monocyte differentiation and acquisition of macrophage-defining cellular features. (A) Schematic representation of the experimental procedure for the analysis of CyaA toxin effects on monocyte differentiation. CD14^+^ monocytes were cultured in DMEM–20 ng/ml M-CSF for 5 days and subjected to mock treatment or exposed for 5 days to 4 ng/ml (22.5 pM) CyaA or to the corresponding noncatalytic CyaA-AC^−^ toxoid, freshly added with every medium change at 24-h intervals. (B) Transmission electron microscopy analysis of the size, pseudopodium production and intracellular organelle accumulation, and complexity of cells after 5 days of differentiation culture with M-CSF. Images are representative of several dozens of cell images inspected for each condition. The CyaA-treated monocytes remained round and small, with no pseudopodia and with reduced organelle content, compared to the mock-treated cells (Control) or toxoid-treated cells (CyaA-AC^−^), which matured into macrophage cells producing numerous pseudopodia and exhibited a high organelle complexity. (C) Cell size and granularity determined by FACS analysis. The low level of side scatter (SSC) corresponds to smaller cell sizes and reduced intracellular organelle complexity. The histogram shown is representative of results from 10 biological replicates.

To investigate the impact of CyaA toxin action on differentiating monocytes, we purified primary human blood monocytes (CD14^+^) from peripheral blood mononuclear cells (PBMCs) of anonymous donors by magnetic separation on CD14 MicroBeads. The obtained cell suspensions contained more than 95% CD14^+^ monocytes (see Fig. S1 in the supplemental material). Cultured for 5 days in Dulbecco’s modified Eagle’s medium (DMEM) supplemented with 20 ng/ml M-CSF (Fig. 1A), the monocytes differentiated into large mature macrophage cells (Fig. 1B) that exhibited such distinguishing characteristics of macrophages as (i) high organelle complexity; (ii) increased numbers of mitochondria, lysosomes, and Golgi bodies; (iii) an expanded ribosome-loaded endoplasmic reticulum; and (iv) numerous pseudopodia (16). Preliminary dose-response experiments (Fig. S2) revealed that prolonged exposure of the isolated monocytes to as little as 4 ng/ml of endotoxin-free CyaA toxin (22.5 pM) was sufficient to cause detectable elevation of cAMP levels (data not shown) but did not affect cell viability, which remained as high as in mock-treated cells (Fig. S3). Monocyte exposure to the activity of 22.5 pM CyaA toxin over 5 days of culture provoked a nearly complete inhibition of M-CSF-induced differentiation of monocytes into macrophages, as judged from the absence of the hallmarks listed above. This blocking effect was clearly due to the cAMP signaling capacity of CyaA. Culture in the presence of the enzymatically inactive CyaA-AC^−^ toxoid, unable to catalyze conversion of ATP to cAMP (17), had no effect on monocyte differentiation. The CyaA toxin-treated cells produced no pseudopodia, their cell volume did not increase over 5 days of culture with M-CSF, and their organelle complexity and level of side scatter remained low (Fig. 1B and C). The cAMP-signaling capacity of CyaA thus blocked the capacity of monocytes to acquire macrophage-defining features.

10.1128/mBio.01743-19.1 FIG S1 Flow cytometry analysis of cells separated from PBMCs on CD14 antibody-coated magnetic beads. The separated cells were stained by fluorescein isothiocyanate (FITC)-labeled anti-CD14 antibody, and a scatter plot is shown. More than 95% of the obtained cells were CD14^+^. Download FIG S1, TIF file, 0.6 MB. Copyright © 2019 Ahmad et al. 2019 Ahmad et al. This content is distributed under the terms of the Creative Commons Attribution 4.0 International license.

10.1128/mBio.01743-19.2 FIG S2 Dose-dependent effects of 5-day exposure to CyaA at the given concentrations were assessed by flow cytometry as median fluorescence intensity (MFI) of cell surface-expressed macrophage markers. CD14^+^ monocytes were cultured for 5 days in DMEM–20 ng/ml M-CSF and the indicated amounts of CyaA. The cells were stained by anti-marker antibodies and analyzed by flow cytometry as described in Materials and Methods. Download FIG S2, TIF file, 0.3 MB. Copyright © 2019 Ahmad et al. 2019 Ahmad et al. This content is distributed under the terms of the Creative Commons Attribution 4.0 International license.

10.1128/mBio.01743-19.3 FIG S3 Viability of cells differentiated in the presence of 20 ng/ml M-CSF and of the indicated compounds for 5 days in DMEM. CD14^+^ monocytes were cultured for 5 days in DMEM–20 ng/ml M-CSF and 22.5 pM of CyaA alone or with the PKA-selective inhibitor Rp-8-Br-cAMPS (0.5 mM) or with CyaA-AC^−^ toxoid or with the PKA-specific activator 6-Bnz-cAMP (100 μM). The cells were then subjected to staining using Hoechst 33258 (1 μg/ml), and cell viability was analyzed by flow cytometry. Download FIG S3, TIF file, 0.1 MB. Copyright © 2019 Ahmad et al. 2019 Ahmad et al. This content is distributed under the terms of the Creative Commons Attribution 4.0 International license.

**FIG 2:**
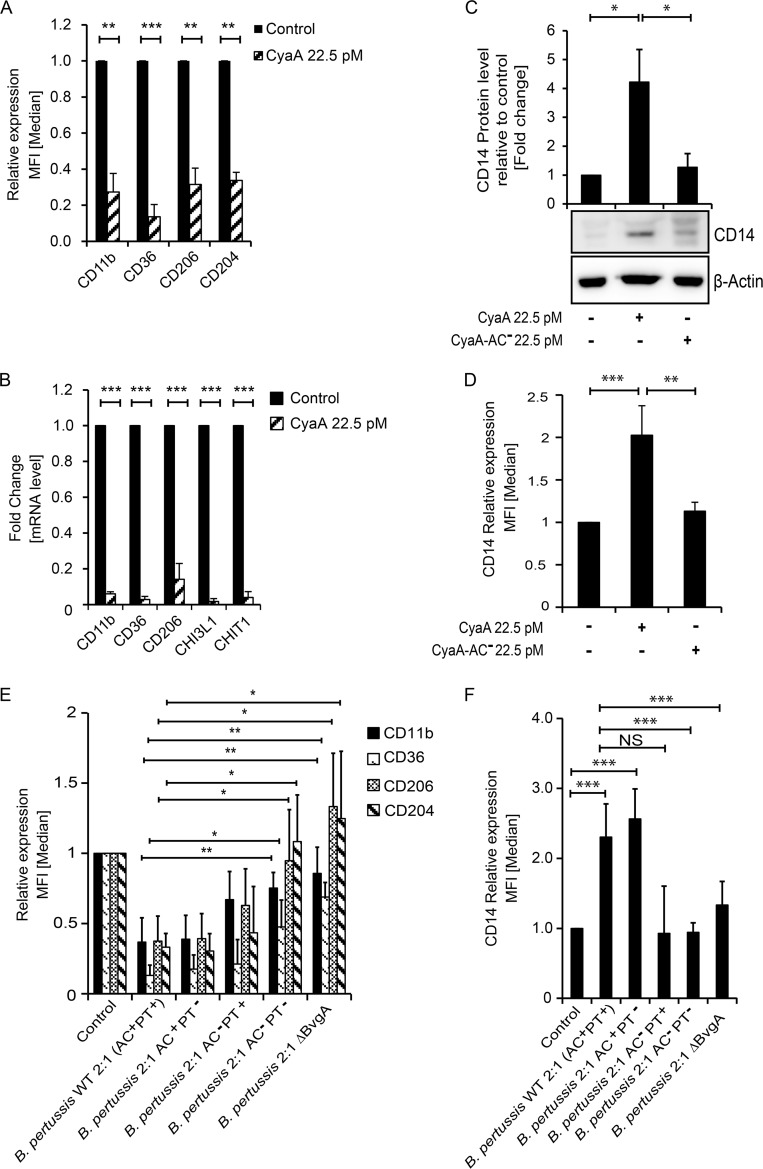
CyaA toxin activity subverts M-CSF-elicited monocyte-macrophage transition. CD14^+^ monocytes were cultured for 5 days in DMEM–20 ng/ml M-CSF and 22.5 pM CyaA or CyaA-AC^−^ (A) FACS analysis of relative levels of cell surface expression of macrophage markers. The median fluorescence intensity (MFI) values shown represent means ± standard deviations (SD). *n* = ≥7; **, *P* < 0.05; ***, *P* < 0.0005. (B) qPCR analysis of transcript levels of macrophage marker genes. The fold change values are means ± SD, *n* ≥ 7 biological replicates performed in duplicates; ***, *P* < 0.0005. (C) CyaA inhibits downregulation of CD14 marker expression on monocytes. Levels of the CD14 marker protein in cellular lysates were analyzed by densitometry of immunoblots of whole-cell lysates and expressed as fold change, assigning the CD14 level of mock-treated cells a value of 1. The values were normalized to the β-actin protein level used as a loading control. Values are means ± SD, *n* = 3; *, *P* < 0.05. (D) Cell surface expression of CD14 detected by flow cytometry. (E) CD14^+^ monocytes were infected by wild-type and mutant strains of B. pertussis CIP 81.32 Tohama I at MOI 2:1 for the first 12 h of culture. Bacteria were next killed by the use of 50 μg/ml polymyxin B plus 50 μg/ml kanamycin for an additional 12 h. At 5 days later, after repeated medium replacement, the expression levels of macrophage markers were analyzed by flow cytometry. (F) CD14^+^ monocytes were treated as described above, and surface expression levels of the CD14 marker were determined by flow cytometry. The MFI values are indicated as means ± SD, *n* ≥ 3 biological replicates; *, *P* < 0.05; **, *P* < 0.005; ***, *P* < 0.0005.

As further determined by flow cytometry and reverse transcription-quantitative PCR (qRT-PCR), the M-CSF-induced upregulation of cell surface expression of characteristic macrophage receptor molecules, such as CD11b, CD36, CD206, and CD204, was inhibited when monocytes were exposed to 22.5 pM CyaA for 5 days in the presence of M-CSF (Fig. 2A). This was due to inhibition of transcription of the macrophage marker genes in the presence of CyaA. The genes specifically upregulated late in macrophage differentiation, such as those encoding CHI3L1 and CHIT1 (18), were not expressed at all in toxin-treated monocytes (Fig. 2B). The monocyte marker CD14 (19, 20) was downregulated in M-CSF-differentiated macrophages cocultured with the CyaA-AC^−^ toxoid. In contrast, the CyaA-produced cAMP blocked downregulation of CD14, which remained expressed in CyaA toxin-treated cells over the 5 days of culture with M-CSF (Fig. 2C), and it was also sorted to the cell surface (Fig. 2D).

Moreover, infection for 12 h with as few as two CyaA-producing B. pertussis bacteria (B. pertussis wild type [WT] = AC^+^ PT^+^ [pertussis toxin positive]) per monocyte cell (MOI 2:1) reproducibly provoked the same inhibitory effect as CyaA addition (Fig. 2E). The inhibition was not due to pertussis toxin (PT) action, and monocyte infection by the B. pertussis AC^+^ PT^−^ mutant secreting a recombinant PT toxoid (21, 22) still blocked the M-CSF-driven differentiation of monocytes into macrophages to the same extent as an infection by the B. pertussis WT bacteria. Hence, the differentiation block was largely due to action of the secreted CyaA toxin. However, among other effects of blocking inhibitory Gα_i/o_ activities in transmission of G-protein-coupled receptor (GPCR) signals, the PT action can also result in elevation of cAMP concentrations in cells, albeit at a lower rate and to a lesser extent (23). Indeed, when monocytes were infected by the B. pertussis AC^−^ PT^+^ bacteria, producing a hemolytic but enzymatically inactive CyaA-AC^−^ toxoid, the secreted PT was able to substitute in part for the action of CyaA (Fig. 2E). In contrast, the inhibition was relieved and the monocytes differentiated and expressed the CD204 and CD206 markers to normal levels when the cells were infected with doubly mutated B. pertussis AC^−^ PT^−^ bacteria not producing active CyaA or PT, as well as upon infection with a B. pertussis ΔBvgA mutant that does not produce any of the known B. pertussis toxins and virulence factors. Intriguingly, the level of expression of CD36 (Fig. 2E) still remained reduced in B. pertussis AC^−^ PT^−^ or B. pertussis ΔBvgA-infected cells, which, in terms of marker levels, showed a greater resemblance to the differentiated macrophages expressing low CD14 levels (Fig. 2F). In contrast, monocytes infected with the B. pertussis WT or the B. pertussis AC^+^ PT^−^ bacteria expressed increased CD14 levels, showing that infection by B. pertussis strains secreting active CyaA toxin hampered M-CSF-driven downregulation of the human monocyte marker CD14.

**FIG 3:**
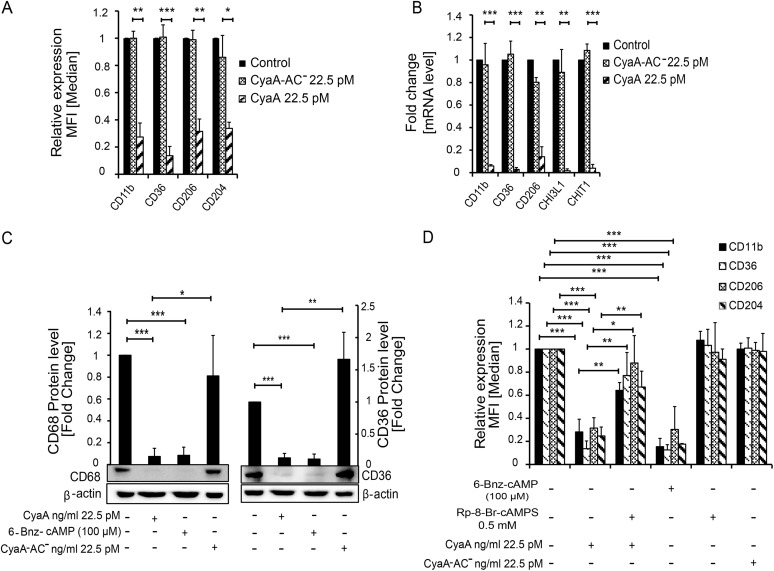
CyaA-produced cAMP signaling mediated through the activity of PKA subverts monocyte-macrophage transition. CD14^+^ monocytes were cultured for 5 days in DMEM–20 ng/ml M-CSF and 22.5 pM CyaA or CyaA-AC^−^ or with the indicated compounds at the given concentrations. (A and B) Suppression of the macrophage marker gene and of surface protein expression depends on the cAMP-producing AC enzyme activity of CyaA. The values are means ± SD, *n* ≥ 7; **, *P* < 0.005; ***, *P* < 0.0005. (C) The cell-permeative and PKA-specific activator 6-Bnz-cAMP (100 μM) suppresses CD36 and CD68 expression to the same level as the cAMP-elevating activity of 22.5 pM CyaA. The CD36 and CD68 proteins were detected by immunoblotting of whole-cell lysates, and their levels were normalized to that of the β-actin loading control. (D) The cell-permeative and PKA-selective inhibitor Rp-8-Br-cAMPS (0.5 mM) blocked CyaA-elicited downregulation of macrophage cell surface markers, whereas the PKA-selective activator 6-Bnz-cAMP (100 μM) mimicked CyaA toxin action and inhibited macrophage marker expression. The values are means ± SD, *n* ≥ 3 biological replicates; *, *P* < 0.05; **, *P* < 0.005; ***, *P* < 0.0005.

The inhibition of monocyte differentiation by CyaA action was entirely due to the capacity of the toxin to hijack cellular signaling by catalyzing conversion of cellular ATP into the second messenger molecule cAMP. Monocytes exposed to an equal concentration of the pore-forming (hemolytic) but catalytically inactive CyaA-AC^−^ toxoid expressed the macrophage differentiation markers at levels comparable to those seen with mock-treated cells (Fig. 3A and B). Furthermore, the toxin action could be fully mimicked by a cell-permeative cAMP analogue that selectively activates protein kinase A (PKA) (24). Exposure of monocytes to 100 μM 6-Bnz-cAMP yielded the same level of suppression of macrophage differentiation marker expression as yielded by the culture of monocytes with 22.5 pM CyaA (Fig. 3C and D; see also Fig. S4). Finally, the inhibition of monocyte differentiation by CyaA toxin could be largely alleviated in the presence of 0.5 mM Rp-8-Br-cAMPS (Fig. 3D), which selectively blocks cAMP signaling through PKA (25). Hence, the inhibition of monocyte differentiation was exclusively mediated by CyaA/cAMP-triggered activation of PKA; the cAMP-activated Epac pathway was not involved.

10.1128/mBio.01743-19.4 FIG S4 CyaA suppresses CD206 expression, and the PKA-specific activator 6-Bnz-cAMP (100 μM) mimicks CyaA action and inhibits the expression of CD206 to the same extent as 22.5 pM CyaA. The CD206 protein was detected by immunoblotting of whole-cell lysates, and its amounts were normalized to the β-actin levels used as the loading control. Download FIG S4, TIF file, 1.0 MB. Copyright © 2019 Ahmad et al. 2019 Ahmad et al. This content is distributed under the terms of the Creative Commons Attribution 4.0 International license.

### CyaA-elicited cAMP signaling prevents acquisition of macrophage functions.

**FIG 4:**
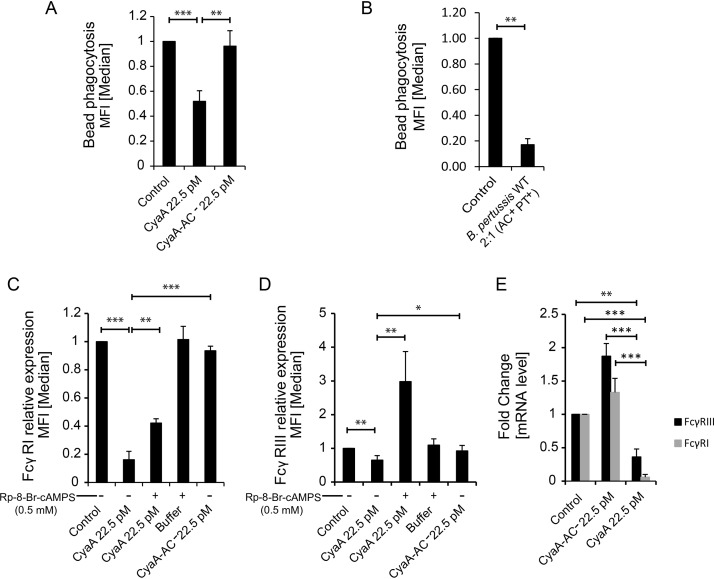
CyaA blocks upregulation of phagocytic capacity. CD14^+^ monocytes were cultured for 5 days in DMEM–20 ng/ml M-CSF and 22.5 pM CyaA or CyaA-AC^−^ or the indicated compounds at the given concentrations. (A and B) Exposure to 4 ng/ml (22.5 pM) CyaA (A) or infection with the B. pertussis WT strain (B. pertussis AC^+^ PT^+^) (B) for 12 h at an MOI of 2:1 suppressed the differentiation-induced increase in the ability to phagocytose serum-opsonized 0.75-μm-diameter fluorescent phagobeads. *n* ≥ 4; **, *P* < 0.05; ***, *P* < 0.005. (C to E) CyaA (22.5 pM)-elicited cAMP signaling inhibited expression of phagocytic receptors FcγRI (C) and FcγRIII (D), and this inhibition was relieved by the activity of PKA-selective inhibitor Rp-8-Br-cAMPS (0.5 mM) (E). *n* ≥ 3 biological replicates; *, *P* < 0.05; **, *P* < 0.005; ***, *P* < 0.0005.

Differentiation of monocytes into macrophages enhances their phagocytic capacity. Tested with normal human serum-opsonized phagobeads, the monocytes cultured with 22.5 pM CyaA exhibited only about 50% of the bead uptake capacity exhibited by the toxoid-treated cells (Fig. 4A). An even stronger inhibition of phagocytic capacity was then observed upon infection of the monocytes with only two B. pertussis bacteria per monocyte cell over 12 h of coculture with M-CSF, prior to the phagocytosis assay (Fig. 4B). Flow cytometry and quantitative PCR (qPCR) analysis revealed that CyaA-exposed monocytes expressed importantly reduced amounts of the phagocytic receptors FcγRI and FcγRIII (*P < *0.005 and *P < *0.005, respectively) due to decreased levels of mRNA transcripts of the corresponding genes (Fig. 4C to E). Here again, the CyaA toxin-triggered inhibition could be alleviated in the presence of Rp-8-Br-cAMPS, the PKA-specific inhibitor of cAMP signaling.

### CyaA provokes dedifferentiation of *in vitro*-differentiated or primary human alveolar macrophages.

**FIG 5:**
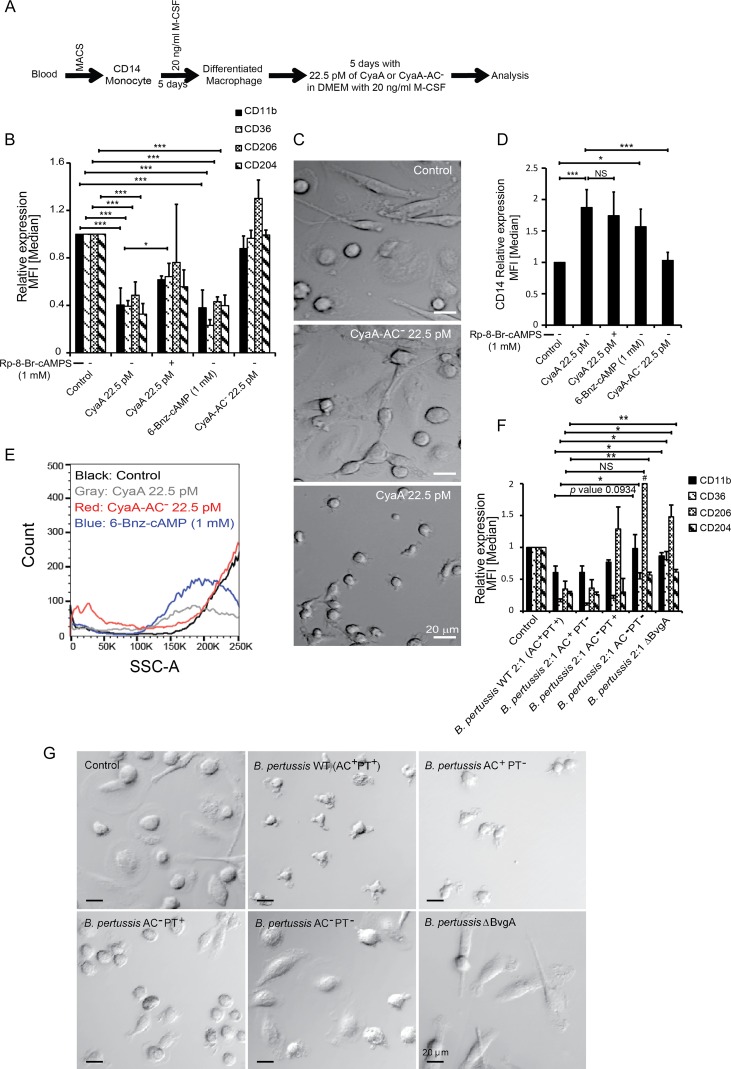
CyaA-elicited cAMP signaling triggers dedifferentiation of fully differentiated macrophages. CD14^+^ monocytes were cultured for 5 days in DMEM–20 ng/ml M-CSF to induce differentiation into macrophages before 22.5 pM CyaA or CyaA-AC^−^ was added, and the culture with 20 ng/ml M-CSF was continued for another 5 days in the presence of CyaA toxin. (A) Schematic depiction of the design of the dedifferentiation experiment. (B) Flow cytometry analysis of cell surface expression of macrophage markers on macrophages exposed for 5 days to CyaA or the indicated compounds. The cell-permeative and PKA-specific activator 6-Bnz-cAMP (1 mM) effectively elicited macrophage dedifferentiation. PKA inhibitor Rp-8-Br-cAMPS (1 mM) partially blocked CyaA toxin-triggered macrophage dedifferentiation. (C) Phase-contrast microscopy images of macrophages that were first differentiated for 5 days with 20 ng/ml M-CSF and then exposed for another 5 days to 22.5 pM CyaA toxin or CyaA-AC^−^ toxoid in the continued presence of 20 ng/ml M-CSF. The shown images are representative of several dozens of images taken in 3 independent experiments (biological replicates). Bar, 20 μm. (D) CyaA-elicited cAMP signaling upregulated CD14 expression in M-CSF-matured macrophages. CyaA-AC^−^ toxoid or mock-treated control cells maintained the CD14^low^ phenotype of differentiated macrophages over 5 days of culture. Upon exposure of M-CSF-differentiated macrophages to the PKA-specific activator 6-Bnz-cAMP (1 mM), the CD14 expression level was upregulated. Exposure to the PKA inhibitor Rp-8-Br-cAMPS (1 mM) did not fully block CyaA toxin-triggered upregulation of CD14. *, *P* < 0.05; **, *P* < 0.005; ***, *P* < 0.0005. (E) M-CSF-differentiated macrophages lost intracellular complexity upon treatment with 22.5 pM CyaA or 1 mM PKA-specific activator 6-Bnz-cAMP for 5 days, as observed by low side scatter. Control and CyaA-AC^−^-exposed cells retained complex cytoplasm rich in cellular organelles. (F) M-CSF-differentiated macrophages were infected by wild-type and mutant strains of B. pertussis CIP 81.32 Tohama I at MOI of 2:1 for the first 12 h of culture. Bacteria were killed by the use of 50 μg/ml polymyxin B plus 50 μg/ml kanamycin over additional 12 h of incubation. 5 days later, after repeated medium replacement, the expression levels of macrophage markers was analyzed by flow cytometry. *n* = 3 biological replicates; *, *P* < 0.05; **, *P* < 0.005; #, above range. (G) Phase-contrast microscopy analysis of cell shape and appearance of mature macrophage cells after 5 days of dedifferentiation resulting following infection with B. pertussis for 12 h at MOI 2:1. Macrophages were first allowed to mature for 5 days with 20 ng/ml M-CSF in DMEM. Next, the macrophage cells were infected with the wild-type strain or the indicated mutant strains of B. pertussis CIP 81.32 Tohama I at MOI of 2:1 for 12 h. The bacteria were then killed with 50 μg/ml polymyxin B plus 50 μg/ml kanamycin over 12 h. The cells were further cultured for 5 days in DMEM–20 ng/ml M-CSF, with half of the media being replenished with fresh medium. After 5 days postinfection, cells were visualized by phase-contrast microscopy, where the macrophage cells exhibited altered morphology, drastic cell shrinkage, and reduced adherence to the surface of the plastic. The shown images are representative of several dozens of images taken on 3 biological replicates. Bar, 20 μm.

The results described above suggest that CyaA action might inhibit transition of infiltrating monocytes to macrophages in B. pertussis-infected airway mucosa. However, the mucosal surface of the airway is patrolled by already mature tissue-resident macrophages and alveolar macrophages. We thus examined if CyaA can trigger dedifferentiation of mature macrophages. Therefore, human monocytes were first differentiated into macrophages in the presence of M-CSF for 5 days and then exposed for another 5 days to 22.5 pM CyaA, or to the CyaA-AC^−^ toxoid, in the continued presence of 20 ng/ml M-CSF (see Fig. 5A). As shown in Fig. 5B and D, the CyaA-AC^−^ toxoid-treated macrophages retained the same level of CD14 and macrophage marker expression as the control cells, retaining the increased size and the characteristic flat and elongated shape of mature macrophage cells (Fig. 5C). In contrast, without decrease of cell viability (Fig. S5), a striking reduction of macrophage marker expression occurred in CyaA-treated cells and treatment with the PKA activator (6-Bnz-cAMP) abrogated the macrophage phenotype as well (Fig. 5B). This process was apparent already on day 3 after CyaA exposure and was completed within 5 days of toxin or 6-Bnz-cAMP addition (data not shown). The macrophage cells regained CD14 levels similar to that of the initial monocytes (Fig. 5D), lost the intracellular complexity (Fig. 5E), and shrank into smaller, round monocyte-like cells over the 5 days of exposure to only 22.5 pM (4 ng/ml) CyaA (see Fig. 5C). The apparent CyaA-triggered macrophage dedifferentiation also occurred upon low-level infection of M-CSF-matured macrophages with B. pertussis WT (AC^+^ PT^+^) bacteria (MOI 2:1). After 12 h of infection, a loss of macrophage marker expression was observed. It was primarily due to CyaA toxin action (Fig. 5F), as infection by B. pertussis AC^+^ PT^−^ bacteria triggered the same level of macrophage marker loss as infection with B. pertussis WT bacteria. Infection with the B. pertussis ΔBvgA mutant, not producing CyaA and PT or any of the known protein toxins of B. pertussis, did not affect the macrophage phenotype; also, the macrophage cells infected by the double mutant B. pertussis AC^−^ PT^−^ strain exhibited characteristics of sham-treated cells that did not dedifferentiate. However, the B. pertussis AC^−^ PT^+^ mutant, producing active pertussis toxin, provoked a partial dedifferentiation of infected macrophage cells, showing that PT action can in part also trigger macrophage dedifferentiation (Fig. 5F). The macrophage cells infected with B. pertussis AC^+^ PT^−^ and B. pertussis AC^−^ PT^+^ bacteria then failed to maintain the morphological features of macrophage cells, namely, the large size and adherence phenotype (Fig. 5G).

**FIG 6:**
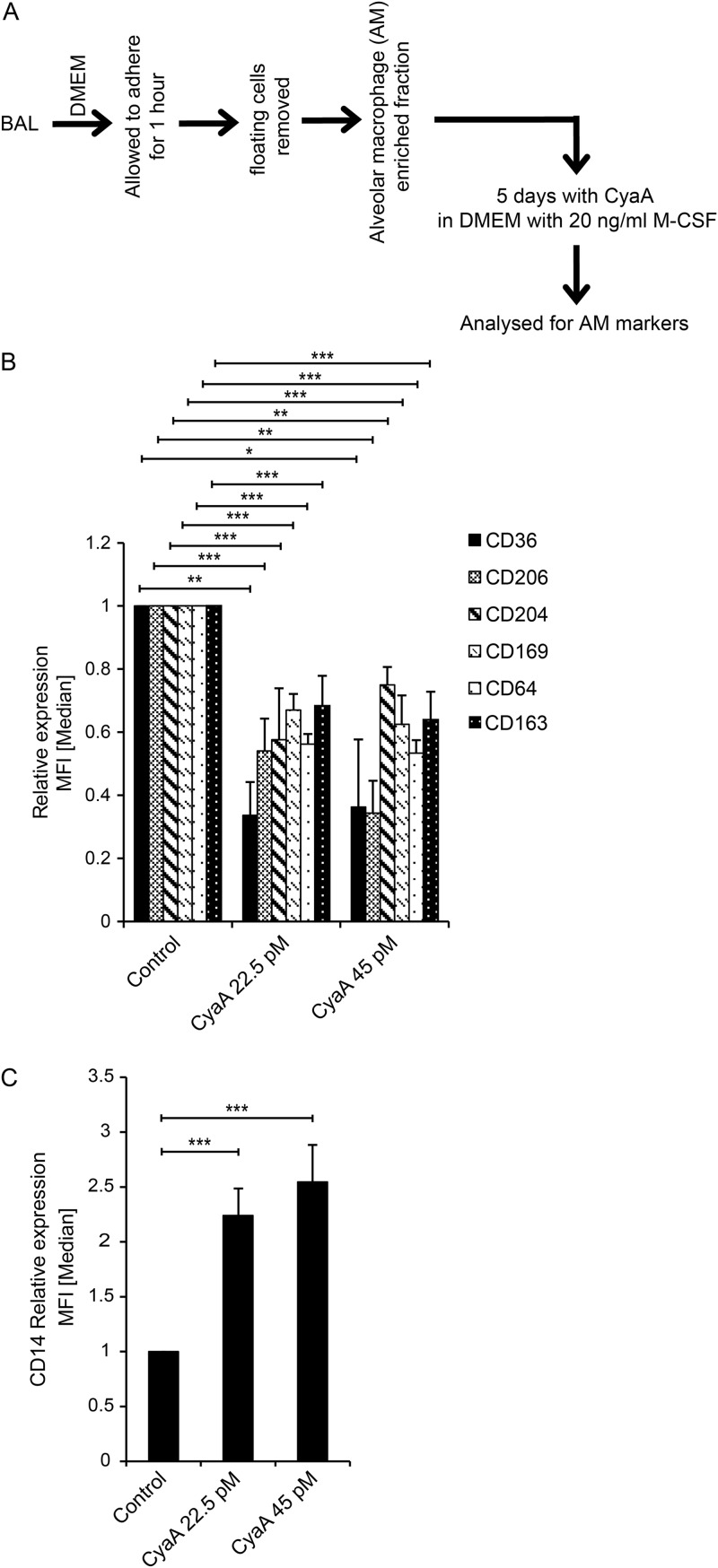
CyaA dedifferentiates human alveolar macrophages. (A) Scheme of the experiment. Alveolar macrophages obtained from human bronchoalveolar lavage (BAL) fluid samples were treated with 4 ng/ml (22.5 pM) or 8 ng/ml (45 pM) of CyaA for 5 days in the presence of M-CSF. (B and C) Macrophage markers (B) and CD14 cell surface expression (C) were analyzed by flow cytometry. *n* = 5 biological replicates; *, *P* < 0.05; **, *P* < 0.005; ***, *P* < 0.0005.

Having found that CyaA can trigger dedifferentiation of *in vitro*-matured macrophage cells, we tested whether CyaA toxin action can also trigger dedifferentiation of terminally differentiated tissue-resident macrophage cells, such as human lung alveolar macrophages. As shown in Fig. 6, when washed adherent cells recovered from bronchoalveolar lavage (BAL) fluid from human patients were exposed to 22.5 pM or 45 pM CyaA toxin for 5 days in the presence of M-CSF (Fig. 6A), a loss of characteristic markers of alveolar macrophages occurred (Fig. 6B). Moreover, the low level of CD14 expression that is typical for alveolar macrophages (20) was reversed and the CyaA-treated cells expressed increased CD14 levels (Fig. 6C).

## DISCUSSION

We found that prolonged *in vitro* exposure of primary human monocytes to as little as ∼22 pM CyaA toxin blocked effectively the M-CSF-triggered differentiation of monocytes into macrophages. Furthermore, those low CyaA amounts triggered sufficient cAMP signaling to promote dedifferentiation of *in vitro* M-CSF-matured or of primary human alveolar macrophages into monocyte-like cells. To the best of our knowledge, this is the first report that tissue-resident macrophages, considered terminally differentiated cells, can be dedifferentiated into monocyte-like cells expressing increased levels of the monocytic marker CD14.

These results go well with our recent observation that the activity of CyaA produced *in vivo* provokes recruitment of inflammatory myeloid cells into B. pertussis-infected mouse lung tissue but that the infiltrating cells exhibited reduced levels of the MHC II molecule on the cell surface (15). It is thus plausible that the capacity of CyaA to block monocyte-to-macrophage transition plays a role in immune evasion of the whooping cough agent, as gradients of the cAMP-elevating adenylate cyclase would form in the B. pertussis*-*infected mucosal tissue at levels that would vary as a function of the distance from the site at which the adhering bacteria are growing. It will be important to determine in the baboon infection model whether CyaA triggers dedifferentiation of the airway mucosa-patrolling or alveolar macrophages in the course of B. pertussis infection.

The capacity of CyaA to block M-CSF signaling-driven downregulation of CD14 expression on monocytes observed here is in line with a previous report indicating the role of cAMP signaling in upregulation of CD14 expression (26). We report that the CyaA-mediated inhibition of monocyte differentiation appears to be entirely mediated by CyaA-elicited cAMP signaling through the protein kinase A (PKA) pathway. The pore-forming but catalytically inactive CyaA-AC^−^ toxoid was unable to suppress monocyte differentiation. Moreover, all of the CyaA-provoked effects could be mimicked by the PKA-selective activator 6-Bnz-cAMP and could be inhibited by the PKA-specific inhibitor Rp-8-Br-cAMPS. This suggests that Epac was not involved. A plausible explanation would be that the cAMP levels produced in cells by the low CyaA amounts (22.5 pM) employed here were insufficient for Epac activation, since Epac exhibits a lower affinity for cAMP than the regulatory subunit of PKA (27, 28). It is worth mentioning that concentrations of the cAMP analogue 6-Bnz-cAMP that were several orders of magnitude higher were needed to achieve the level of inhibition of monocyte differentiation (0.1 mM) and macrophage dedifferentiation (1 mM). This may have several reasons, such as a physical interaction of the AC polypeptide with some cytosolic proteins (e.g., calmodulin) or the incapacity of 6-Bnz-cAMP to trigger influx of calcium ions. This accompanies membrane translocation of the AC enzyme of CyaA and might support the cAMP signaling activity of CyaA. More likely, the higher 6-Bnz-cAMP concentrations, in the range typically used in PKA signaling studies, were needed because of the diffusion barrier of the cell membrane that limits cell entry of the compound. Further, the extremely high specific enzymatic activity of the AC enzyme of CyaA that translocates across the cholesterol-rich membrane microdomains (lipid rafts) would generate high localized gradients of cAMP right in the submembrane space of the cell where PKA anchored through A-kinase anchoring proteins (AKAPs) amplifies and propagates the cAMP signal through the downstream pathway (4). Such an effect may require increased concentrations of a compound that has to permeate from outside the cell.

The inflammatory monocytes represent the second most predominant type of cells after the neutrophils that infiltrate the infected mucosa (29). In the function of the external signals received at the mucosal surface, the monocytes differentiate into macrophages and, importantly, contribute to clearing of pathogens by opsonophagocytic killing and by secretion of the cytokines that shape the development of the adaptive immune response. Inhibition of monocyte maturation by CyaA would thus represent an important mechanism by which B. pertussis may subvert host immunity. Here, we show that monocytes exposed for 5 days to sublethal but cAMP-signaling amounts of CyaA were locked in the monocytic state and failed to differentiate into macrophages. In line with the observed reduced level of expression of phagocytic receptors, CyaA-treated monocytes exhibited reduced phagocytic capacity, few pseudopodia, and a lack of the organelle complexity characteristic of macrophages (30).

Several bacterial pathogens employ manipulation of cAMP levels in host cells as a strategy for evasion of host immune defenses (31–33). For example, ExoY of Pseudomonas aeruginosa, the edema factor of Bacillus anthracis, the cholera toxin of Vibrio cholerae, and the heat-labile enterotoxin from Escherichia coli all represent toxins that elevate cytosolic cAMP levels in various host cell types. In the function of the respective levels of produced cAMP, all these toxins might exploit a mechanism similar to that employed by B. pertussis CyaA to trigger suppression of monocyte differentiation. B. pertussis would then express a whole set of toxins and virulence factors, among which the most notorious are the pertussis toxin (PT) and the CyaA toxin, which both manipulate cellular cAMP levels, albeit to quite different extents. Unlike CyaA, PT is not an AC enzyme itself and exerts complex systemic effects through inactivation of the inhibitory Gα_i/o_ subunit of the heterotrimeric G proteins in various cell types. This ablates the various functions of the Gα_i/o_ proteins and blocks signaling of the Gi protein-coupled receptors (GiPCRs) through Gα_i/o_, with upregulation of cellular cAMP levels in leukocytes due to absence of GiPCR-driven inhibition of the endogenous AC enzyme. Among the complex manifestations of PT action are the leukocytosis typical for pertussis disease and the inhibition of neutrophil extravasation and migration into infected tissue in the early phase of B. pertussis infection (34, 35). Following those functions, CyaA would then decrease phagocyte activity levels in the infected tissue both through direct inhibition of the Syk signaling required for activation of the opsonophagocytic process (36) and through the inhibition of differentiation of infiltrating inflammatory monocytes into macrophages observed here. It is plausible that the actions of PT and CyaA *in vivo* are complementary and synergic, accomplishing immunosuppressive subversion of host defense and thereby enabling B. pertussis infection of the airway mucosa (37). Arresting differentiation of infiltrating monocytes into macrophages by the action of CyaA would likely represent an important colonization advantage for B. pertussis, as (i) monocytes are less potent in bactericidal activities than macrophages and (ii) monocytes differentiated into macrophages acquire a self-renewal capacity. Preventing monocyte-to-macrophage transition at the site of infection is thus likely to play a role in the colonization capacity of B. pertussis. To the best of our knowledge, the data presented here represent the first report that differentiated macrophage cells can be dedifferentiated back to monocyte-like cells. Reprogramming of infiltrating monocytes or of alveolar macrophages to less bactericidal and short-living monocyte-like cell types would further empower the bacteria to resist the sentinel immune cells. Such a toxin-mediated capacity of B. pertussis would be particularly pertinent to infection of infants and elderly, whose immune defense relies mainly on innate immunity, with monocytes and macrophages playing a significant protective role.

In this study, we used M-CSF to promote monocyte differentiation because M-CSF is produced in most tissues and its secretion recruits inflammatory cells to the site of infection or injury *in vivo* (38, 39). M-CSF is detectable in normal human sera at levels of up to 10 ng/ml (40, 41). Its production is upregulated in response to bacterial endotoxin and Toll-like receptor (TLR) signaling during a Gram-negative bacterial infection of the lungs to the point that lungs become the major site of M-CSF production (42, 43). It is noteworthy that abnormal expression of M-CSF was observed in various cancer cells and that this is used as an effective marker in diagnosing of ovarian and breast cancers (44–46). Our data suggest that targeted manipulation of cAMP levels might have an effect in treatment of some malignant tumors. For example, it is tempting to speculate that CyaA, applied at very low and possibly not even immunogenic concentrations (20 pM), might induce dedifferentiation, shrinkage, and metabolic reprogramming of CD11b-expressing myeloid cancer cells. It was reported recently that manipulation of PKA activity by adjustment of cAMP levels can promote the mesenchymal cell-to-epithelial cell transition of certain tumor-initiating cells, thus reducing their metastatic potential and rendering them more susceptible to chemotherapy (47). It thus appears worth exploring whether CyaA could do the same *in vivo*, with myeloma cells expressing the CD11b/CD18 receptor exploited by CyaA for binding to phagocytic cells.

In summary, our findings provide a new insight into the capacity of B. pertussis-produced CyaA toxin to paralyze the bactericidal functions of the sentinel cells of the innate immune system. These observations suggest a possible role of CyaA in arresting of monocyte-to-macrophage differentiation of inflammatory monocytes in B. pertussis-infected mucosa *in vivo*. Reprogramming of airway-resident and alveolar macrophages to revert to a less bactericidal monocyte phenotype would then represent an unprecedented mechanism of immune evasion by a pathogen.

## MATERIALS AND METHODS

### Antibodies (Abs) and reagents.

Anti-CD11b (ICRF44), anti-CD36 (TR9), FcγRIII (3G8), FcγRI (10.1), CD206 (15-2), anti-CD169 (7-239), anti-CD163 (GHI/61), and anti-CD14 (MEM18) were purchased from Exbio (Czech Republic). Anti-CD204-APC (anti-CD204-allophycocyanin) (PSL204) was purchased from Invitrogen, CD206-PE-Cy7 (CD206-phycoerythrin-Cy7) (19.2) from eBioscience, anti-CD68 (298807) from R&D Systems, and anti-CD206 (C10) from Santa Cruz Biotechnology. RNA blue was purchased from TopBio (Czech Republic), Fluoresbrite phagobeads from Polysciences (catalog no. 17153), recombinant human M-CSF from Peprotech (catalog no. 300-25), saponin from Sigma (catalog no. 47036), and human CD14 MicroBeads from Miltenyi Biotec. Albumin fraction V was purchased from Carl Roth (catalog no. 8076). Rp-8Br-cAMPS and 6-Bnz-cAMP were purchased from Biolog (Germany), and antibiotic antimycotic solution (100×) was purchased from Sigma.

### CyaA and CyaA-AC^−^ purification.

CyaA and CyaA-AC^−^ were produced in E. coli strain XL1-Blue (Stratagene, La Jolla, CA) expressing *cyaC* and *cyaA* genes from the pT7CACT1 plasmid (17). Proteins were purified by a combination of ion-exchange chromatography performed using DEAE-Sepharose and hydrophobic chromatography performed using phenyl-Sepharose, as described previously (17). Endotoxin-free toxin was prepared by washing the toxin-bound resin with 60% isopropanol (48). The purified proteins contained less than 0.1 endotoxin units (EU) of lipopolysaccharide per 1 μg of protein, as determined using a chromogenic limulus amebocyte lysate assay kit (QCL-1000; Lonza, Walkersville, MD).

### Bordetella pertussis strains and infection conditions.

B. pertussis strains were derived from the Tohama I strain obtained from the Institute Pasteur collection (CIP 81.32). Bacteria were grown at 37°C on Bordet-Gengou agar (Difco, USA) supplemented with 1% glycerol and 15% defibrinated sheep blood in a humidified 5% CO_2_ atmosphere for 5 days. Liquid subcultures were grown for 18 h at 37°C in modified Stainer-Scholte medium supplemented with 5 g/liter of Casamino Acids (Difco) and 0.5 g/liter of Heptakis(2,6-di-O-methyl)-beta-cyclodextrin (Sigma-Aldrich) until an *A*_600_ value of ∼1 (3 × 10^9^ CFU/ml) was reached. The suspension was diluted 1:10 in fresh medium, and 2 × 10^6^ CFU of B. pertussis (10 μl) was added to 10^6^ monocytes or macrophage cells mixed in 1 ml of DMEM without antibiotics, as indicated in the figure legends.

B. pertussis mutants were constructed using allelic exchange as described previously (49). The pSS4245 suicide vector was a kind gift of Scott Stibitz (U.S. CBER, FDA). Two different recombinant plasmids were constructed by insertion of PCR-amplified fragments using appropriate primers. Site-directed mutations were introduced into the *ptxS1* gene by PCR mutagenesis. Briefly, PCR fragments of chromosomal DNA were inserted into pSS4245 vector using the NotI and BamHI sites. The first construct, named pSS4245mutPtxS1 (R9K E129G), contained a *ptxS1* gene fragment harboring the codon mismatch CGC→AAG, causing the R9K substitution, and the codon mismatch GAA→GGG, causing the E129G substitution, thus resulting in production of a genetically detoxified PT toxoid (50). The second construct, pSS4245mutCyaA (188GS189), contained insertions of Gly (GGA) and Ser (TCC) codons between codons 188 and 189 of the *cyaA* open reading frame, resulting in production of an enzymatically inactive adenylate cyclase toxoid (CyaA-AC^−^) as previously described (15, 17). The mutated sequences were marked by silent mutations introducing restriction sites for the BsrGI, EcoRV, and BamHI enzymes, which enabled straightforward verification of the presence of mutations by PCR analysis.

To construct the double B. pertussis mutant, the mutations in the *cyaA* and *ptxS1* genes were successively introduced into the bacterial chromosome by allelic exchange and were verified by resequencing of the corresponding PCR-amplified gene fragment. First, the mutation in *ptx* gene (R9K E129G), yielding the inactive PT toxin, was introduced, followed by mutagenesis of the *cyaA* gene (188GS189), yielding CyaA-AC^−^. The newly created B. pertussis mutants were named B. pertussis CIP 81.32mutCyaA (B. pertussis AC^−^ PT^+^), B. pertussis CIP 81.32mutPtxS1 (B. pertussis AC^+^ PT^−^), and B. pertussis CIP 81.32 mutPtxS1+mutCyaA (B. pertussis AC^−^ PT^−^). Production of CyaA and PT and of their respective toxoids by the used strains was analyzed by Western blotting of whole bacterial cell lysates, using a monoclonal antibody (MAb) specific for CyaA (51) and a polyclonal serum raised against the recombinant S1 subunit of PT. The Tohama I-derived B. pertussis strain with a deletion of the *bvgA* gene (B. pertussis ΔBvgA) was described previously (52) and was a kind gift of B. Vecerek of the Institute of Microbiology of the CAS in Prague.

### Monocyte separation.

Human peripheral blood mononuclear cells (PBMCs) were purified from blood leukopacks from anonymous healthy donors and were purchased at the Thomayer Hospital in Prague, Czech Republic. Cells were concentrated using density gradient centrifugation over Ficoll-Paque media (GE Healthcare) according to the Miltenyi Biotec protocol, and CD14^+^ monocytes were separated from PBMCs using magnetically activated cell sorting (MACS; Miltenyi Biotec) with CD14 MicroBeads. The cell pellet was resuspended in 0.5 ml phosphate-buffered saline (PBS)–bovine serum albumin (PBS–BSA), and a 25-μl volume of MicroBeads conjugated to monoclonal anti-human CD14 antibody (Miltenyi Biotec) was added. After 20 min on ice, the cell pellet was washed with 10 ml of PBS-BSA, centrifuged, and resuspended in 2 ml PBS-BSA. Labeled cells were separated using the Possel program (Miltenyi Biotec). The purity of the eluted CD14^+^ monocytes exceeded 90%, as verified by fluorescence-activated cell sorter (FACS) analysis.

### Monocyte differentiation and toxin treatment.

CD14^+^ cells were seeded into 12-well plates (10^6^/ml per well) in DMEM with 10% fetal calf serum (FCS) containing 20 ng/ml of recombinant human M-CSF (Peprotech) and incubated for 5 days at 37°C with or without CyaA toxin (22.5 pM). When applicable, cells were preincubated for 1 h with Rp-8-Br-cAMPS prior to addition of toxin or 6-Bnz-cAMP at the indicated concentrations. Prior to CyaA toxin addition, the cells were replaced every 24 h into fresh DMEM containing 20 ng/ml M-CSF and the indicated toxin or activator/inhibitor supplements were added at the indicated concentrations. After 5 days of culture, with or without toxin, the cells were washed with ice-cold PBS (pH 7.4) and resuspended in Hanks’ balanced salt solution-FCS (HBSS-FCS). It was systematically verified by staining with Hoechst 33258 (1 μg/ml) that culture with CyaA toxin or with the activators/inhibitors did not affect the viability of the cells compared to mock-treated cells (see Fig. S3 in the supplemental material). Typically, more than 80% of the cells remained viable after 5 days of *in vitro* culture with M-CSF with or without 22.5 pM CyaA toxin. For FACS analysis, the cells were stained with the indicated directly labeled MAbs for 40 min at 4°C in 50 μl HBSS-FCS buffer according to the manufacturer’s instructions. After washes were performed with 1 ml HBSS-FCS, cells were resuspended in HBSS-FCS containing 1 μg/ml of Hoechst 33258 and analyzed by flow cytometry. Data were analyzed using FlowJo software (Tree Star, Ashland, OR).

10.1128/mBio.01743-19.5 FIG S5 Viability of dedifferentiated cells. CD14^+^ monocytes were allowed to differentiate for 5 days in DMEM–20 ng/ml M-CSF. Next, the differentiated cells were treated for another 5 days with 22.5 pM of CyaA alone or with selective PKA inhibitor Rp-8-Br-cAMPS (1 mM) or with mutant CyaA-AC^−^ toxoid or with PKA-specific activator 6-Bnz-cAMP (1 mM). The cells were then subjected to staining using Hoechst 33258 (1 μg/ml), and cell viability was analyzed by flow cytometry. Download FIG S5, TIF file, 2.9 MB. Copyright © 2019 Ahmad et al. 2019 Ahmad et al. This content is distributed under the terms of the Creative Commons Attribution 4.0 International license.

For immunoblotting, the cells were lysed with 10 mM Tris (pH 7.4), 140 mM NaCl, 1 mM EDTA, 1% NP-40, 0.1% SDS, 1 mM phenylmethylsulfonyl fluoride (PMSF), and cOmplete mini protease inhibitor cocktail (Roche), probed with appropriate target-specific primary Abs (1:1,000), decorated with horseradish peroxidase (HRP)-conjugated secondary Abs (1:5,000), and detected with enhanced chemiluminescent West Femto maximum sensitivity substrate (Pierce) using a GBOX-Chemi-XRQ-E system (Syngene, Frederick, MD, USA) and densitometric quantification in ImageJ (https://imagej.nih.gov/ij/).

Detection of the intracellular CD68 molecule by anti-hCD68 was carried out as per the instructions of the manufacturer (R&D Systems).

### Bacterial infection of CD14^+^ monocytes and of CD14^low^ macrophage cells.

Purified CD14^+^ monocytic cells, or the CD14^low^ macrophages that have been differentiated with 20 ng/ml M-CSF for 5 days, were seeded into 12-well plates in DMEM–20 ng/ml M-CSF and infected with the wild-type B. pertussis CIP 81.32 Tohama I strain or its corresponding mutants at a multiplicity of infection (MOI) of 2 bacteria per cell (2:1). The culture plates were incubated for 12 h at 37°C in a humidified 5% CO_2_ atmosphere. After 12 h, the bacteria were killed by replacing half of the media with fresh DMEM containing 20 ng/ml M-CSF and 50 μg/ml of polymyxin B (PMB) plus 50 μg/ml of kanamycin and incubated for another 12 h. Next, every 24 h, half of the medium was replaced with fresh DMEM–20 ng/ml M-CSF and with the antibiotic cocktail (Sigma). After 5 days of culture, the cells were stained with antibodies and analyzed by flow cytometry for monocytes/macrophage markers and/or for uptake of fluorescent phagobeads.

### Phagocytosis assay.

The phagocytic potency of cells was determined using 0.75-μm-diameter phagobeads (Fluoresbrite YG Microspheres [catalog no. 17153]; Polysciences Inc., Warrington, PA) according to the manufacturer’s instructions (technical datasheet 430).

### Macrophage dedifferentiation analysis.

Ethical approval for the use of alveolar macrophages of anonymous donors indicated for diagnostic bronchial lavage was obtained from the Ethics Committee of the Institute of Clinical and Experimental Medicine and Thomayer Hospital in Prague (docket no. A-18-20). The donors gave informed consent for research use of the leftovers of the cells contained in the lavage fluid samples. Briefly, the lavage fluid was mixed 1:1 with prewarmed DMEM and centrifuged at 1,300 rpm for 3 min at room temperature. The cell pellet was resuspended in DMEM containing a cocktail of antibiotics (Sigma) and allowed to settle and adhere for 1 h to untreated plastic petri dishes. Floating cells were removed by gentle washing, and the adherent cells were utilized for the dedifferentiation experiment.

Monocytes differentiated for 5 days into macrophages in the presence of 20 ng/ml M-CSF (see above), or the primary human alveolar macrophages purified as described above, were cocultured with 4 ng/ml (22.5 pM) CyaA toxin, or the noncatalytic CyaA-AC^−^ toxoid in the continued presence of 20 ng/ml M-CSF for additional 5 days, before the expression of surface markers was assessed by flow cytometry.

### Quantitative real-time PCR.

Total cellular RNA was isolated using RNA Blue (TopBio, Czech Republic) and treated with DNase I (New England Biolabs) at 37°C for 1 h. Purified RNA (1 μg) was reverse transcribed using a high-capacity cDNA reverse transcription kit (Applied Biosystems) according to manufacturer’s instructions. Triplicate qRT-PCRs (10-μl reaction mixture volume) were run in HOT FIREPol EvaGreen qPCR Supermix (Solis Biodyne) with 0.8 μM primers (see Table S1 in the supplemental material) by the use of an initial step performed at 95°C for 5 min followed by 40 cycles (95°C for 15 s, 60°C for 20 s, and 72°C for 30 s), using a Bio-Rad CFX384 real-time PCR system. The threshold cycle (2^−ΔΔ^*^CT^*) values were determined, and the mRNA levels were normalized to that of human β-2 microglobulin mRNA.

10.1128/mBio.01743-19.6 TABLE S1 List of primers used for analysis of gene expression by qPCR. Download Table S1, PDF file, 0.04 MB. Copyright © 2019 Ahmad et al. 2019 Ahmad et al. This content is distributed under the terms of the Creative Commons Attribution 4.0 International license.

### Determination of cAMP concentrations.

Monocytes were exposed to 22.5 pM of CyaA for 3 h, and intracellular cAMP levels were quantified by enzyme-linked immunosorbent assay (ELISA) as previously described (53).

### Electron microscopy.

CD14-positive cells were treated with 22.5 pM CyaA toxin or CyaA-AC^−^ toxoid for 5 days as described above and fixed for 2 h with 2% glutaraldehyde–PBS. After three washes with ice-cold PBS, cells were postfixed with 0.5% osmium tetroxide–PBS and incubated overnight at 4°C. Fixed and washed samples were dehydrated with ethanol using a standard procedure. Cells were embedded in epoxy resin (EMBed-812 embedding kit; Electron Microscopy Sciences). Ultrathin sections were visually contrasted using lead citrate and uranyl acetate (54). Final samples were examined using an FEI Morgagni 268(D) electron microscope (FEI, Brno, Czech Republic) at 80 kV. Digital images were recorded with a MegaViewIII slow-scan camera and processed by AnalySis 3.2 software (Olympus Soft Imaging Solutions GmbH, Münster, Germany) using standard software modules (shading correction, digital contrast enhancement).

### Statistics.

For statistical analysis, a paired *t* test was used.

## References

[1] Vojtova J, Kamanova J, Sebo P 2006 *Bordetella* adenylate cyclase toxin: a swift saboteur of host defense. Curr Opin Microbiol 9:69–75. doi:10.1016/j.mib.2005.12.011. 16406775

[2] Linhartova I, Bumba L, Masin J, Basler M, Osicka R, Kamanova J, Prochazkova K, Adkins I, Hejnova-Holubova J, Sadilkova L, Morova J, Sebo P 2010 RTX proteins: a highly diverse family secreted by a common mechanism. FEMS Microbiol Rev 34:1076–1112. doi:10.1111/j.1574-6976.2010.00231.x. 20528947 PMC3034196

[3] Guermonprez P, Khelef N, Blouin E, Rieu P, Ricciardi-Castagnoli P, Guiso N, Ladant D, Leclerc C 2001 The adenylate cyclase toxin of *Bordetella pertussis* binds to target cells via the alpha(M)beta(2) integrin (CD11b/CD18). J Exp Med 193:1035–1044. doi:10.1084/jem.193.9.1035. 11342588 PMC2193436

[4] Bumba L, Masin J, Fiser R, Sebo P 2010 *Bordetella* adenylate cyclase toxin mobilizes its beta2 integrin receptor into lipid rafts to accomplish translocation across target cell membrane in two steps. PLoS Pathog 6:e1000901. doi:10.1371/journal.ppat.1000901. 20485565 PMC2869314

[5] Morova J, Osicka R, Masin J, Sebo P 2008 RTX cytotoxins recognize beta2 integrin receptors through N-linked oligosaccharides. Proc Natl Acad Sci U S A 105:5355–5360. doi:10.1073/pnas.0711400105. 18375764 PMC2291121

[6] Guo Q, Shen Y, Lee YS, Gibbs CS, Mrksich M, Tang WJ 2005 Structural basis for the interaction of *Bordetella pertussis* adenylyl cyclase toxin with calmodulin. EMBO J 24:3190–3201. doi:10.1038/sj.emboj.7600800. 16138079 PMC1224690

[7] Wolff J, Cook GH, Goldhammer AR, Berkowitz SA 1980 Calmodulin activates prokaryotic adenylate cyclase. Proc Natl Acad Sci U S A 77:3841–3844. doi:10.1073/pnas.77.7.3841. 6253992 PMC349722

[8] Kamanova J, Kofronova O, Masin J, Genth H, Vojtova J, Linhartova I, Benada O, Just I, Sebo P 2008 Adenylate cyclase toxin subverts phagocyte function by RhoA inhibition and unproductive ruffling. J Immunol 181:5587–5597. doi:10.4049/jimmunol.181.8.5587. 18832717

[9] Confer DL, Eaton JW 1982 Phagocyte impotence caused by an invasive bacterial adenylate cyclase. Science 217:948–950. doi:10.1126/science.6287574. 6287574

[10] Cerny O, Anderson KE, Stephens LR, Hawkins PT, Sebo P 2017 cAMP signaling of adenylate cyclase toxin blocks the oxidative burst of neutrophils through Epac-mediated inhibition of phospholipase C activity. J Immunol 198:1285–1296. doi:10.4049/jimmunol.1601309. 28039302

[11] Cerny O, Kamanova J, Masin J, Bibova I, Skopova K, Sebo P 2015 *Bordetella pertussis* adenylate cyclase toxin blocks induction of bactericidal nitric oxide in macrophages through cAMP-dependent activation of the SHP-1 phosphatase. J Immunol 194:4901–4913. doi:10.4049/jimmunol.1402941. 25876760

[12] Ahmad JN, Cerny O, Linhartova I, Masin J, Osicka R, Sebo P 2016 cAMP signalling of *Bordetella* adenylate cyclase toxin through the SHP-1 phosphatase activates the BimEL-Bax pro-apoptotic cascade in phagocytes. Cell Microbiol 18:384–398. doi:10.1111/cmi.12519. 26334669

[13] Khelef N, Zychlinsky A, Guiso N 1993 *Bordetella pertussis* induces apoptosis in macrophages: role of adenylate cyclase-hemolysin. Infect Immun 61:4064–4071. 8406793 10.1128/iai.61.10.4064-4071.1993 PMC281125

[14] Ecker J, Liebisch G, Englmaier M, Grandl M, Robenek H, Schmitz G 2010 Induction of fatty acid synthesis is a key requirement for phagocytic differentiation of human monocytes. Proc Natl Acad Sci U S A 107:7817–7822. doi:10.1073/pnas.0912059107. 20385828 PMC2867858

[15] Skopova K, Tomalova B, Kanchev I, Rossmann P, Svedova M, Adkins I, Bibova I, Tomala J, Masin J, Guiso N, Osicka R, Sedlacek R, Kovar M, Sebo P 2017 Cyclic AMP-elevating capacity of adenylate cyclase toxin-hemolysin is sufficient for lung infection but not for full virulence of *Bordetella pertussis*. Infect Immun 85:e00937-16. doi:10.1128/IAI.00937-16. 28396322 PMC5442630

[16] Jakubzick CV, Randolph GJ, Henson PM 2017 Monocyte differentiation and antigen-presenting functions. Nat Rev Immunol 17:349–362. doi:10.1038/nri.2017.28. 28436425

[17] Osicka R, Osickova A, Basar T, Guermonprez P, Rojas M, Leclerc C, Sebo P 2000 Delivery of CD8(+) T-cell epitopes into major histocompatibility complex class I antigen presentation pathway by *Bordetella pertussis* adenylate cyclase: delineation of cell invasive structures and permissive insertion sites. Infect Immun 68:247–256. doi:10.1128/IAI.68.1.247-256.2000. 10603395 PMC97128

[18] Krause SW, Rehli M, Kreutz M, Schwarzfischer L, Paulauskis JD, Andreesen R 1996 Differential screening identifies genetic markers of monocyte to macrophage maturation. J Leukoc Biol 60:540–545. doi:10.1002/jlb.60.4.540. 8864140

[19] Gantner F, Kupferschmidt R, Schudt C, Wendel A, Hatzelmann A 1997 In vitro differentiation of human monocytes to macrophages: change of PDE profile and its relationship to suppression of tumour necrosis factor-alpha release by PDE inhibitors. Br J Pharmacol 121:221–231. doi:10.1038/sj.bjp.0701124. 9154331 PMC1564680

[20] Passlick B, Flieger D, Ziegler-Heitbrock HW 1989 Identification and characterization of a novel monocyte subpopulation in human peripheral blood. Blood 74:2527–2534. 2478233

[21] Burnette WN, Cieplak W, Mar VL, Kaljot KT, Sato H, Keith JM 1988 Pertussis toxin S1 mutant with reduced enzyme activity and a conserved protective epitope. Science 242:72–74. doi:10.1126/science.2459776. 2459776

[22] Cockle SA 1989 Identification of an active-site residue in subunit S1 of pertussis toxin by photocrosslinking to NAD. FEBS Lett 249:329–332. doi:10.1016/0014-5793(89)80652-0. 2737291

[23] Locht C, Coutte L, Mielcarek N 2011 The ins and outs of pertussis toxin. FEBS J 278:4668–4682. doi:10.1111/j.1742-4658.2011.08237.x. 21740523

[24] Christensen AE, Selheim F, de Rooij J, Dremier S, Schwede F, Dao KK, Martinez A, Maenhaut C, Bos JL, Genieser H-G, Døskeland SO 2003 cAMP analog mapping of Epac1 and cAMP kinase. Discriminating analogs demonstrate that Epac and cAMP kinase act synergistically to promote PC-12 cell neurite extension. J Biol Chem 278:35394–35402. doi:10.1074/jbc.M302179200. 12819211

[25] Gjertsen BT, Mellgren G, Otten A, Maronde E, Genieser HG, Jastorff B, Vintermyr OK, McKnight GS, Doskeland SO 1995 Novel (Rp)-cAMPS analogs as tools for inhibition of cAMP-kinase in cell culture. Basal cAMP-kinase activity modulates interleukin-1 beta action. J Biol Chem 270:20599–20607. doi:10.1074/jbc.270.35.20599. 7657638

[26] Liu S, Morris SM Jr, Nie S, Shapiro RA, Billiar TR 2000 cAMP induces CD14 expression in murine macrophages via increased transcription. J Leukoc Biol 67:894–901. doi:10.1002/jlb.67.6.894. 10857864

[27] Purves GI, Kamishima T, Davies LM, Quayle JM, Dart C 2009 Exchange protein activated by cAMP (Epac) mediates cAMP-dependent but protein kinase A-insensitive modulation of vascular ATP-sensitive potassium channels. J Physiol 587:3639–3650. doi:10.1113/jphysiol.2009.173534. 19491242 PMC2742287

[28] de Rooij J, Rehmann H, van Triest M, Cool RH, Wittinghofer A, Bos JL 2000 Mechanism of regulation of the Epac family of cAMP-dependent RapGEFs. J Biol Chem 275:20829–20836. doi:10.1074/jbc.M001113200. 10777494

[29] Shi C, Pamer EG 2011 Monocyte recruitment during infection and inflammation. Nat Rev Immunol 11:762–774. doi:10.1038/nri3070. 21984070 PMC3947780

[30] Rougerie P, Miskolci V, Cox D 2013 Generation of membrane structures during phagocytosis and chemotaxis of macrophages: role and regulation of the actin cytoskeleton. Immunol Rev 256:222–239. doi:10.1111/imr.12118. 24117824 PMC3806206

[31] Sarantis H, Grinstein S 2012 Subversion of phagocytosis for pathogen survival. Cell Host Microbe 12:419–431. doi:10.1016/j.chom.2012.09.001. 23084912

[32] Sansonetti PJ, Di Santo JP 2007 Debugging how bacteria manipulate the immune response. Immunity 26:149–161. doi:10.1016/j.immuni.2007.02.004. 17307704

[33] McDonough KA, Rodriguez A 2012 The myriad roles of cyclic AMP in microbial pathogens: from signal to sword. Nat Rev Microbiol 10:27–38. doi:10.1038/nrmicro2688. PMC3785115 22080930

[34] Morse SI, Morse JH 1976 Isolation and properties of the leukocytosis- and lymphocytosis-promoting factor of *Bordetella pertussis*. J Exp Med 143:1483–1502. doi:10.1084/jem.143.6.1483. 58054 PMC2190226

[35] Andreasen C, Carbonetti NH 2008 Pertussis toxin inhibits early chemokine production to delay neutrophil recruitment in response to *Bordetella pertussis* respiratory tract infection in mice. Infect Immun 76:5139–5148. doi:10.1128/IAI.00895-08. 18765723 PMC2573337

[36] Osicka R, Osickova A, Hasan S, Bumba L, Cerny J, Sebo P 2015 *Bordetella* adenylate cyclase toxin is a unique ligand of the integrin complement receptor 3. Elife 4:e10766. doi:10.7554/eLife.10766. 26650353 PMC4755762

[37] Melvin JA, Scheller EV, Miller JF, Cotter PA 2014 *Bordetella pertussis* pathogenesis: current and future challenges. Nat Rev Microbiol 12:274–288. doi:10.1038/nrmicro3235. 24608338 PMC4205565

[38] Mantovani A 1994 Tumor-associated macrophages in neoplastic progression: a paradigm for the in vivo function of chemokines. Lab Invest 71:5–16. 7518882

[39] Pollard JW 2004 Tumour-educated macrophages promote tumour progression and metastasis. Nat Rev Cancer 4:71–78. doi:10.1038/nrc1256. 14708027

[40] Hume DA, MacDonald KP 2012 Therapeutic applications of macrophage colony-stimulating factor-1 (CSF-1) and antagonists of CSF-1 receptor (CSF-1R) signaling. Blood 119:1810–1820. doi:10.1182/blood-2011-09-379214. 22186992

[41] Cebon J, Layton JE, Maher D, Morstyn G 1994 Endogenous haemopoietic growth factors in neutropenia and infection. Br J Haematol 86:265–274. doi:10.1111/j.1365-2141.1994.tb04725.x. 7515265

[42] Bettina A, Zhang Z, Michels K, Cagnina RE, Vincent IS, Burdick MD, Kadl A, Mehrad B 2016 M-CSF mediates host defense during bacterial pneumonia by promoting the survival of lung and liver mononuclear phagocytes. J Immunol 196:5047–5055. doi:10.4049/jimmunol.1600306. 27183631 PMC4893984

[43] Nelson S, Daifuku R, Andresen J 1994 Use of filgrastim (r-metHuGCSF) in infectious diseases, p 253–266. *In* Morstyn G, Dexter TM (ed), Filgrastim (r-metHuG-CSF) in clinical practice. Dekker, New York, NY.

[44] Lin EY, Gouon-Evans V, Nguyen AV, Pollard JW 2002 The macrophage growth factor CSF-1 in mammary gland development and tumor progression. J Mammary Gland Biol Neoplasia 7:147–162. doi:10.1023/A:1020399802795. 12465600

[45] Smith HO, Anderson PS, Kuo DY, Goldberg GL, DeVictoria CL, Boocock CA, Jones JG, Runowicz CD, Stanley ER, Pollard JW 1995 The role of colony-stimulating factor 1 and its receptor in the etiopathogenesis of endometrial adenocarcinoma. Clin Cancer Res 1:313–325. 9815987

[46] Kacinski BM 1997 CSF-1 and its receptor in breast carcinomas and neoplasms of the female reproductive tract. Mol Reprod Dev 46:71–74. doi:10.1002/(SICI)1098-2795(199701)46:1<71::AID-MRD11>3.0.CO;2-6. 8981366

[47] Pattabiraman DR, Bierie B, Kober KI, Thiru P, Krall JA, Zill C, Reinhardt F, Tam WL, Weinberg RA 2016 Activation of PKA leads to mesenchymal-to-epithelial transition and loss of tumor-initiating ability. Science 351:aad3680. doi:10.1126/science.aad3680. 26941323 PMC5131720

[48] Franken KL, Hiemstra HS, van Meijgaarden KE, Subronto Y, den Hartigh J, Ottenhoff TH, Drijfhout JW 2000 Purification of his-tagged proteins by immobilized chelate affinity chromatography: the benefits from the use of organic solvent. Protein Expr Purif 18:95–99. doi:10.1006/prep.1999.1162. 10648174

[49] Stibitz S 1994 Use of conditionally counterselectable suicide vectors for allelic exchange. Methods Enzymol 235:458–465. doi:10.1016/0076-6879(94)35161-9. 8057916

[50] Buasri W, Impoolsup A, Boonchird C, Luengchaichawange A, Prompiboon P, Petre J, Panbangred W 2012 Construction of *Bordetella pertussis* strains with enhanced production of genetically-inactivated pertussis toxin and pertactin by unmarked allelic exchange. BMC Microbiol 12:61. doi:10.1186/1471-2180-12-61. 22524455 PMC3349578

[51] Lee SJ, Gray MC, Guo L, Sebo P, Hewlett EL 1999 Epitope mapping of monoclonal antibodies against *Bordetella pertussis* adenylate cyclase toxin. Infect Immun 67:2090–2095. 10225859 10.1128/iai.67.5.2090-2095.1999 PMC115942

[52] Keidel K, Amman F, Bibova I, Drzmisek J, Benes V, Hot D, Vecerek B 2018 Signal transduction-dependent small regulatory RNA is involved in glutamate metabolism of the human pathogen *Bordetella pertussi*s. RNA 24:1530–1541. doi:10.1261/rna.067306.118. 30097543 PMC6191719

[53] Karimova G, Pidoux J, Ullmann A, Ladant D 1998 A bacterial two-hybrid system based on a reconstituted signal transduction pathway. Proc Natl Acad Sci U S A 95:5752–5756. doi:10.1073/pnas.95.10.5752. 9576956 PMC20451

[54] Reynolds ES 1963 The use of lead citrate at high pH as an electron-opaque stain in electron microscopy. J Cell Biol 17:208–212. doi:10.1083/jcb.17.1.208. 13986422 PMC2106263

