# Supplementary figures and images for: Bordetella Adenylate Cyclase Toxin Inhibits Monocyte-to-Macrophage Transition and Dedifferentiates Human Alveolar Macrophages into Monocyte-like Cells
Source: mBio. 2019 Sep 24;10(5):e01743-19. doi: 10.1128/mBio.01743-19 (PMC6759761; doi:10.1128/mBio.01743-19)

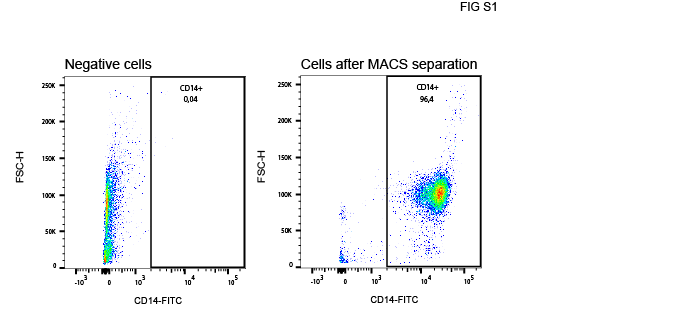

Supplement: FIG S1 [file mBio.01743-19-sf001.tif]

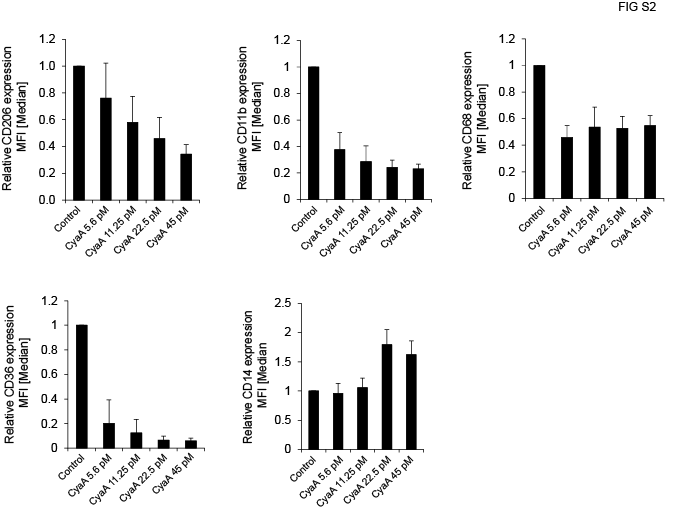

Supplement: FIG S2 [file mBio.01743-19-sf002.tif]

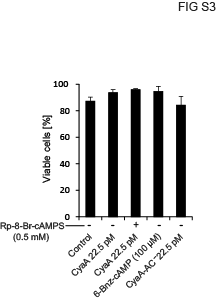

Supplement: FIG S3 [file mBio.01743-19-sf003.tif]

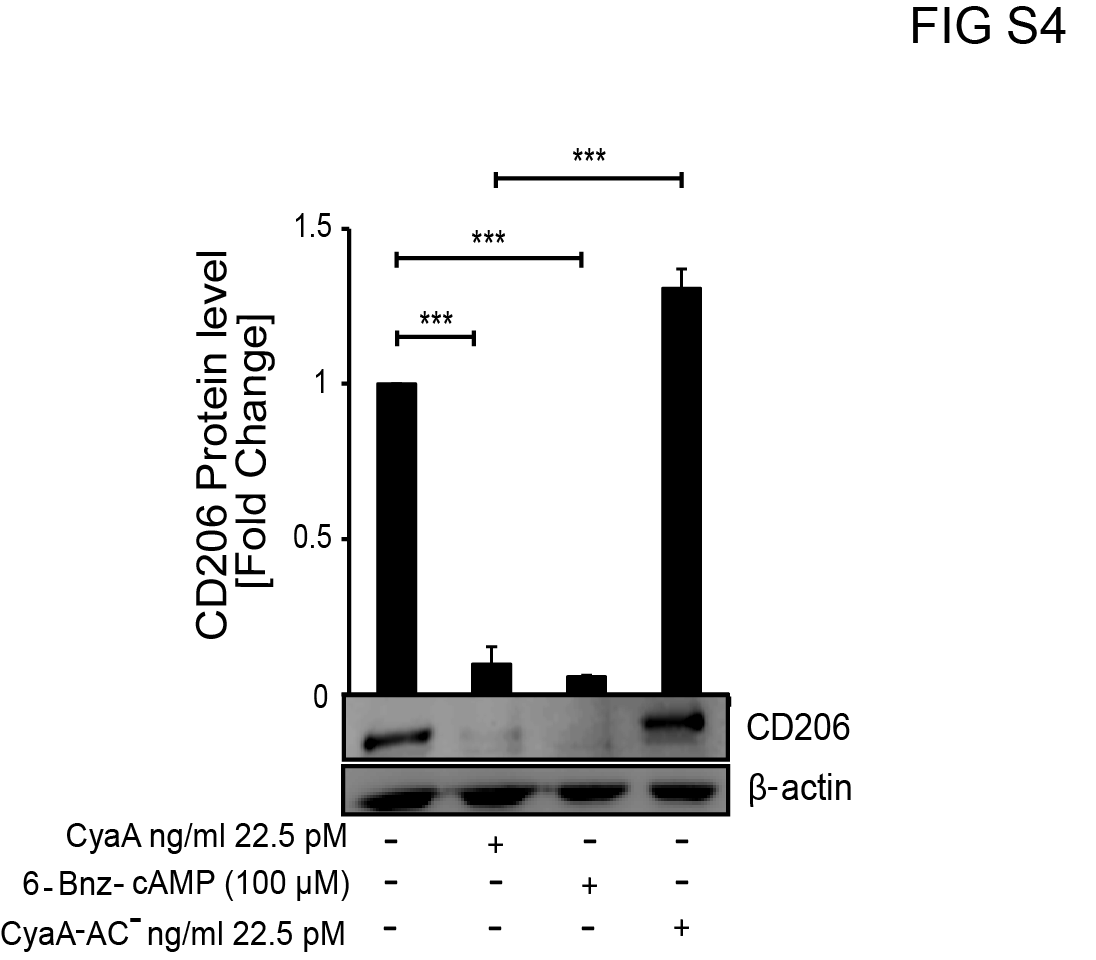

Supplement: FIG S4 [file mBio.01743-19-sf004.tif]

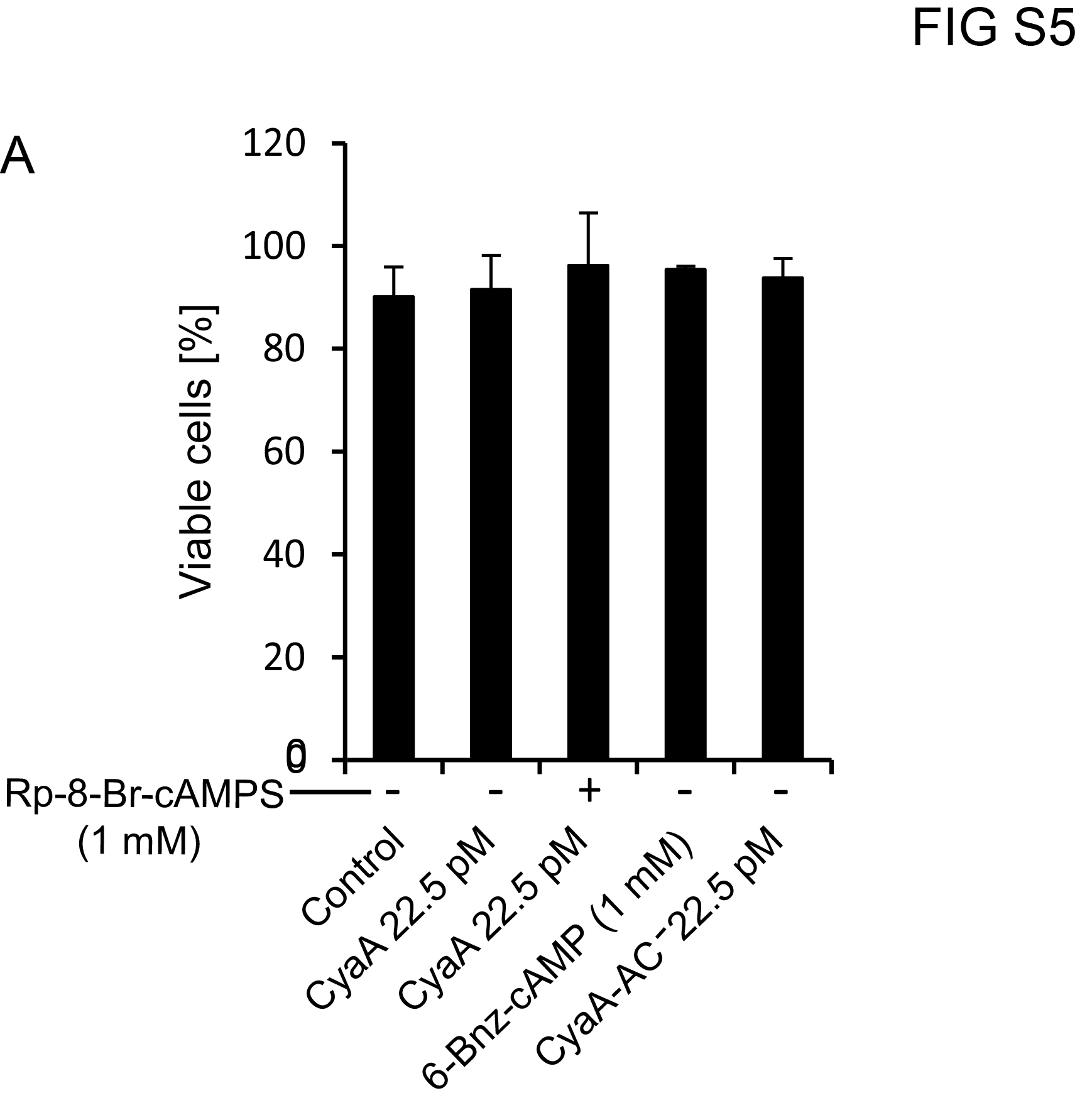

Supplement: FIG S5 [file mBio.01743-19-sf005.tif]
