# Supplementary material for: Bordetella Adenylate Cyclase Toxin Inhibits Monocyte-to-Macrophage Transition and Dedifferentiates Human Alveolar Macrophages into Monocyte-like Cells
Source: mBio. 2019 Sep 24;10(5):e01743-19. doi: 10.1128/mBio.01743-19 (PMC6759761; doi:10.1128/mBio.01743-19)
Supplement: TABLE S1 [file mBio.01743-19-st001.pdf]

TABLE S1

List of primers used for analysis of gene expression by qPCR

Transcript	Forward primer	Reverse primer	Amplicon size (bp)	CCDS (NCBI)
CD11b	GGGAAGTGGCAAGGAATGTA	CTGCGTGTGCTGTTCTTTGT	214	54004.1
CD36	GCTGTCATTGGTGCTGTCCTGG	GCTGCTGTTCATCATCACTTCCTGTGG	204	34673.1
CD206	GTGGCACCAGGCGAGGAAAAG	CGGTCACTCCACTGCCAACC	168	7123.2
CHIT1	GGTCTGCTACTTCACCAACTGGG	GGCCATTGAACTCCTGGTAGAGAGTC	176	1436.1
CHI3L1	CTGCTACTACACCAGCTGGTCC	GAGACCCAAAGTTCCATCCTCCG	236	1435.1
FcγRI	GTTCCAGTTGATGGGCAAGT	TCTGGCACCTGTATTCACCA	229	933.1
FcγRIII	TGAGGTGTCACAGCTGGAAG	GGTTGACACTGCCAAACCTT	218	41433.1
